# A randomized pilot trial on the effect of granulocyte-colony stimulating factor on antibody response in hemodialysis patients who had not responded to routine hepatitis B virus vaccine

**DOI:** 10.12860/jnp.2015.03

**Published:** 2015-01-01

**Authors:** Jamshid Roozbeh, Kamran Bagheri-Lankarani, Poopak Mohaghegh, Ghanbarali Raeesjalali, Saeed Behzadi, Mehdi Sagheb, Mehrdad Vossoughi, Bahar Bastani

**Affiliations:** ^1^Shiraz Nephro-Urology Research Center, Shiraz University of Medical Sciences, Shiraz, Iran; ^2^Department of Internal Medicine, Shiraz University of Medical Sciences, Shiraz, Iran; ^3^Health Policy Research Center, Shiraz University of Medical Sciences, Shiraz, Iran; ^4^Department of Dental Public Health, School of Dentistry, Shiraz University of Medical Sciences, Shiraz, Iran; ^5^Division of Nephrology, Saint Louis University, School of Medicine, Saint Louis, Missouri, USA

**Keywords:** Granulocyte-colony stimulating factor, Hemodialysis, Vaccination, Hepatitis B, Granulocyte-macrophage colonystimulating

## Abstract

*Background:* Various strategies have been applied to improve the response to hepatitis B virus (HBV) vaccination in hemodialysis patients.

*Objectives:* The present study was under taken to compare the seroconversion rate of hemodialysis patients who had not respond to 3 intramuscular (IM) doses (40 μg each) of HBV vaccine , after a fourth IM dose (40 μg) of HBV vaccine that was administered alone or with subcutaneous granulocyte-colony stimulating factor (G-CSF) (5 μg/kg).

*Patients and Methods:* Twenty six hemodialysis patients who had not responded to 3 IM injections of HBV vaccine were randomized into 2 groups: Group 1 received a booster dose of 40 μg HBV vaccine IM, group 2 received a booster dose of 40 μg HBV vaccine IM plus 5 μg/kg subcutaneous G-CSF. Antibody to hepatitis B surface antigen was measured 1 month after the booster dose.

*Results:* Seroconversion rate in group 1 was 40%. There was a trend towards a higher seroconversion rate at 60% in group 2 patients; however, because of the small number of patients it did not reach statistical significance.

*Conclusions:* Larger number of patients and other innovative strategies should be applied for vaccination of this group of patients. More prolonged follow up of the patients is needed to evaluate the duration of protection induced by each method of vaccination.

**Implication for health policy/practice/research/medical education:**

This study was performed on 26 hemodialysis patients who had not responded to three intramuscular (IM) injections of hepatitis B virus (HBV) vaccine. The patients were divided into two groups: Group 1 received a booster dose of 40 μg HBV vaccine IM, group 2 received a booster dose of 40 μg HBV vaccine IM plus 5 μg/kg subcutaneous granulocyte-colony stimulating factor (G-CSF). There was a trend towards a higher seroconversion rate (60% vs 40%) in group 2 compared to group 1 patients, however, because of the small number of patients it did not reach statistical significance. Larger number of patients should be applied to better find the impact of G-CSF treatment on intensification of seroconversion rate in hemodialysis patients.

## 1. Background

Patients with end stage renal disease who are maintained on hemodialysis are at increased risk of hepatitis B infection (1-3). Unlike healthy adults, patients undergoing dialysis who are infected with hepatitis B virus (HBV) usually have a mild and asymptomatic disease (2). However, up to 80% of them become chronic carriers of the virus, and most remain highly infectious, as indicated by a high prevalence of the hepatitis B surface antigenemia and hepatitis Be antigenemia (4,5). HBV infection becomes chronic in 30-60% of hemodialysis patients, as compared with less than 10% in non-uremic patients (6). Center for disease control (CDC) currently recommends that all dialysis patients should receive HBV vaccine (7). However, only 50% to 60% of dialysis patients develop sufficient hepatitis B surface antibodies (HBS-Ab) after recombinant HBV vaccine, as compared to over 90% response rate in patients who do not have renal failure (8-11).

Renal failure is associated with an acquired immunodeficiency state, as evidenced by reduced cellular immune in-vitro and in-vivo responses (12-15). Non-responsiveness to HBV vaccination was found to be associated with reduced interleukin-2 (IL-2) production by T-lymphocytes (14), which is essential for generating activated antigen-specific T-cells required for B-cell activation and antibody production (14). Nevertheless, a case-control study found that hemodialysis patients vaccinated against HBV had a 70% lower risk for infection as compared with non-vaccinated patients (16).

Various strategies have been applied to improve the response to HBV vaccination in hemodialysis patients. These include, doubling the dose of vaccine (i.e., 40 µg per dose), increasing vaccine frequency (Heptavax 40 µg intramuscular (IM) at 0, 1, and 6 months or Engerix 40 µg IM at 0, 1, 2 and 6 months), administering the vaccine in deltoid muscle or intradermally (7,8,10,11,17-21) or by adding immuno-stimulants or adjuvants, e.g., thymopectin, AS04, or GM-CSF (granulocyte-monocyte colony-stimulating factor) or G-CSF (granulocyte-colony stimulating factor) (22-29).

## 2. Objectives

The present study was performed to compare the seroconversion rate of hemodialysis patients who had not respond to three IM doses (40 µg each) of HBV vaccine (Heber Biovac, HerberBiotec, Havana, Cuba), after a fourth IM dose (40 µg) of HBV vaccine that was administered alone or with subcutaneous G-CSF (5 µg/kg).

## 3. Patients and Methods

### 3.1. Study patients

This study followed an earlier study by Roozbeh et al. (18) where 62 newly diagnosed end-stage renal failure (ESRD) patients who upon initial screening were found to have negative serology for hepatitis B surface antigen (HBS-Ag), hepatitis B surface antibody (HBS-Ab) and hepatitis B core antibody (HBC-Ab) received 3 IM doses of HBV vaccination. In 46 patients, HBS-Ab titer was checked 6 months after the last vaccine dose by an enzyme-linked immunosorbent assay (ELISA) technique (Diapro, Italy). Twenty-six (56.5%) patients did not respond to vaccination (HBS-Ab titer <10 IU/L). In the present prospective randomized clinical trial, these 26 patients were randomly divided into two groups: Group 1 (n=13) received another IM dose of 40 µg of recombinant HBV vaccine in deltoid muscle, group 2 (n=13) received the same dose vaccine in deltoid muscle plus 5 µg/kg of G-CSF (Neupogen, Roche) subcutaneously on the same side. A well-trained nurse in the hemodialysis center conducted all vaccinations.

HBS-Ab titer was measured by ELISA technique in 23 of the 26 patients, 1 month after the last dose of vaccine. Three patients were lost in follow up. HBS-Ab titer equal or greater than 10 IU/L was regarded as positive. The response rate was compared between the two groups.

The patients’ age, body mass index (BMI) [weight/(height)2], serum albumin level, and underlying renal disease status were also recorded.

### 3.2. Ethical issues

1) The research followed the tenets of the Declaration of Helsinki; 2) informed consent was obtained; and 3) the research was approved by the ethical committee of Shiraz University of Medical Sciences.

### 3.2. Statistical analysis

Quantitative data were described using mean± SD, and qualitative data were summarized using frequencies. Chi-square and Fisher’s exact tests were used to compare sex ratio and response rate (level >10 IU/L for HBS-Ab titer) between the two groups. Kolmogorov-Smirnov test was employed to evaluate normality assumption for qualitative data. We used Student’s t-test to compare mean of quantitative variables between the groups. Data analysis was used SPSS software. P value of <0.05 was considered statistically significant.

## 4. Results

There were no significant differences in mean age, BMI, gender, and primary renal diseases between the two groups (all p>0.05). Tables 1 and 2 summarize the patients’ data.

**Table 1:** Demographic and clinical characteristics

	Group 1 HBV vaccination	Group 2 HBV vaccination + G-CSF	P
Age (years)	48.9 ± 15.8	53.3 ± 15.1	0.5
BMI (kg/m^2^)	22.9 ± 2.6	23.7 ± 2.4	0.6
Albumin (g/dL)	3.6 ± 0.7	4.0 ± 0.5	0.4
Sex (male/female)	10/3	10/3	1.0
Developing protective level (+/-)*	4/9	8/5	0.2

*HBS-Ab titer > 10 IU/L was considered as developing protective level (+) and values less than 10 IU/L was considered as negative (-).

**Table 2:** Primary renal diseases

Type of renal disease	Group 1 HBV vaccination	Group 2 HBV vaccination + G-CSF
Hypertension	3	4
Diabetic nephropathy	4	6
Infection	3	1
Glomerulonephritis	1	-
Unknown	2	2
Total	13	13

In group 1, three patients were lost at one month follow up, and thus, HBS-Ab titer was checked 1 month after vaccination in only 10 patients in group 1 and 13 patients in group 2.

Four (40%) of the 10 patients in group 1, and 8 (60%) of the 13 patients in group 2 developed protective level (>10 IU/L) of HBS-Ab titer. Due to small number of patients in each group this did not reach statistical significance (p= 0.2).

## 5. Discussion

The standard three doses of HBV vaccine at 0, 1 and 6 months result in a protective antibody response to HBV surface antigen (anti-HBS-Ab > 10 IU/ml) in more than 90% of healthy people (30-32). Half of the non-responders develop protective antibody titer after revaccination with high dose HBV vaccine (3 doses, 40 mcg IM each) or standard dose (3 doses, 10 mcg IM each, immediately after GM-CSF 125 mcg) (33). However, only 50-60% of dialysis patients develop sufficient anti-HBS-Ab after recombinant HBV vaccination (8-11).

GM-CSF and G-CSF are immunomodulatory cytokines. To date only a few studies have used GM-CSF as HBV vaccine adjuvant in healthy people, or have investigated the efficacy and safety of a single dose of GM-CSF as an adjuvant in healthy non-responders (33). GM-CSF has also been investigated as an adjuvant therapy to enhance HBV vaccine response in hemodialysis patients in primary vaccination (24-29). Kapoor et al. noted a significant increase in seroconversion rate (100% vs. 44%), and mean HBS-Ab titer (70 vs. 22 IU/L) 1 month after 4 doses (40 µg each) of HBV vaccine (Engerix-B) plus one dose of GM-CSF (3 µg/kg) as compared to 4 doses of vaccine alone (25).

Amanda et al. showed a much higher response rate in hemodialysis patients who received three doses of HBV vaccine (40 mcg each, given in deltoid muscle) 24 hours after one dose of GM-CSF (4-5 mcg/kg, given subcutaneously) as compared to patients who received vaccine alone (83% vs. 33%) (26). They also showed that in hemodialysis patients who had failed standard double-dose HBV vaccine, a booster dose of HBV vaccine 24 hours after a dose of GM-CSF significantly increased response rate as compared with a booster dose vaccine alone (87.5% vs. 25%) (26). Hess et al. studied the efficacy of one dose of 0.5 µg/kg, 5 µg/kg or 10 µg/kg GM-CSF, administered subcutaneously 24 hours prior to a booster IM dose of 40 µg HBV vaccine in non-responder hemodialysis patients (i.e., had not responded to at least 3 standard vaccinations of 40 mcg HBV vaccine) and found 46.7% seroconversion rate 4 weeks after the booster dose vaccine. The best response rate (80%) was seen with 5 µg/kg GM-CSF (27). However, Evans et al. found no significant difference in seroconversion rate at day 21 when a single dose of 40 µg or 80 µg GM-CSF or placebo was administered with a 40 µg HBV vaccine in hemodialysis patients who had not seroconverted after at least 3 doses of recombinant HBV vaccine (28). The lower dose of GM-CSF (0.5-1 mcg/kg) used in that study was administered IM at the time of injection of HBV vaccine, rather than subcutaneously 24 hours earlier, and this may explain the lack of its efficacy (28).

The rationale for using GM-CSF lies in its multiple effects on the immune system, which include macrophage activation, increasing MHC class II antigen expression, enhancing memory cell generation via T and B cell activation, enhancing cell maturation and migration, increasing the number of circulating monocytes and enhancing their differentiation to professional antigen presenting cells, and enhancing dendritic cell maturation (especially type 2 dendritic cells) (29-34). However, in a study which non-responder hemodialysis patients were given two additional booster vaccines, and both preceded by administration of GM-CSF the day before, the GM-CSF was an effective adjuvant for HBV vaccination while it paradoxically decreased the antigen presenting capacity of peripheral blood mononuclear cells and the number of circulating dendritic cells (29).

Moreover, in a randomized, placebo-controlled trial, subcutaneous administration of GM-CSF, as a vaccine adjuvant, at the time of vaccination did not augment the antibody response to influenza or hepatitis A, or cellular response to tetanus and diphtheria toxoid in healthy volunteers (35,36). Despite these contradictory results on the mechanism of action of GM-CSF, a recent meta-analysis has favored GM-CSF as compared to controls, and showed a significant dose/response effect of GM-CSF (37).

In contrast to GM-CSF, G-CSF is a lineage specific colony-stimulating factor. While it mainly affects neutrophils, it also affect antigen presenting cells (35). There is only one report of successfully using G-CSF (Neupogen) as a vaccine adjuvant in an individual who had previously failed three courses of conventional vaccination (38). Moreover, G-CSF has been suggested to be better tolerated than GM-CSF (39).

Our study is unique in that we used G-CSF (Neupogen) as adjuvant to HBV vaccine in previously non-responder hemodialysis patients. In our study the seroconversion rate in non-responder hemodialysis patients, following 40 µg HBV vaccine plus 5 µg/kg subcutaneous G-CSF was 60% compared to 40%, if the booster dose of HBV vaccine was administered alone. The lack of statistically significant difference in response rates of the 2 groups could be due to the small number of patients studied. Moreover, the application of G-CSF was not associated with any adverse events.

## 6. Conclusions

In summary, prospective studies with larger number of patients, and using G-CSF or other innovative methods to enhance antibody response to HBV vaccination are needed. Moreover, longer follow up of the patients is needed to evaluate the duration of protection induced by each vaccination strategy.

## Acknowledgments

The authors appreciate generous free supply of G-CSF (Neupogen) by Amgen pharmaceuticals to perform this study.

## Authors’ contributions

All authors wrote the paper equally.

## Conflict of interests

The authors declared no competing interests.

## Funding/Support

None.
